# Unveiling hotspots of emerging research in the miRNA-related mechanism underlying cancer through comprehensive bibliometric analysis with implications for precision medicine and non-invasive diagnostics

**DOI:** 10.3389/fonc.2024.1521251

**Published:** 2025-01-15

**Authors:** Zhirui Zhang, Wenhuan Song, Wenyu Chen, Wenze Cui, Wenyi Chen, Qinheng Zhang, Wenwen Ji, Yinglin Wang, Jiayi Wang, Wenhao Yu, Mingkun Yu, Tao Hao, Hong Jiang

**Affiliations:** ^1^ Binzhou Medical University Hospital, Binzhou Medical University, Binzhou, Shandong, China; ^2^ College of Traditional Chinese Medicine, Binzhou Medical University, Yantai, Shandong, China; ^3^ Binzhou Medical College Affiliated Traditional Chinese Medicine Hospital, Binzhou, Shandong, China; ^4^ Shandong University of Traditional Chinese Medicine, University Science Park, Jinan, Shandong, China; ^5^ Department of Colorectal Hernia Surgery, Binzhou Medical University Hospital, Binzhou, Shandong, China

**Keywords:** microRNA, cancer diagnosis, bibliometric analysis, non-invasive diagnostics, personalized medicine

## Abstract

**Background and objective:**

MicroRNAs (miRNAs) are implicated in cancer by exerting roles in tumor growth, metastasis, and even drug resistance. The general trends of miRNA research in diverse cancers are not fully understood. In this work, miRNA-related research in colorectal cancer, prostate cancer, leukemia, and brain tumors was analyzed in search of key research trends with clinical potential.

**Methods:**

A bibliometric analysis of articles, spanning from 2014 to 2024, was carried out with the major focus laid on four types of cancers. The Co-citation network analysis, keyword bursts, and the collaborative pattern were done in VOSviewer and CiteSpace, respectively.

**Results:**

Colorectal cancer had the highest publication volume, with research primarily focusing on gene expression, extracellular vesicles, and non-coding RNAs. Prostate cancer showed a shift toward clinical applications, while leukemia and brain tumor research, though less extensive, highlighted miRNA’s potential in early diagnosis and treatment. Co-citation analysis identified emerging research collaborations and key contributors.

**Conclusion:**

miRNA plays a pivotal role in cancer diagnosis, biomarker development, and therapeutic interventions. With advancements in non-invasive diagnostics and personalized medicine, miRNA offers significant potential for clinical applications. Future research should focus on miRNA’s role in drug resistance and combination therapies to accelerate its clinical translation.

## 1 Introduction

Cancer is a multifaceted disease that involves both genetic and epigenetic changes. MicroRNAs (miRNAs) play a vital role in tumorigenesis, progression, metastasis, and drug resistance by regulating oncogenes and tumor suppressor genes, thus influencing various pathological processes. As endogenous non-coding RNAs, miRNAs suppress the expression of multiple target genes, impacting a range of physiological and pathological mechanisms (1). Aberrant miRNA expression is linked to almost all types of malignancies, functioning either as oncogenes or tumor suppressors during tumorigenesis (2). Moreover, miRNAs regulate essential cancer cell processes, such as the cell cycle, DNA damage response, migration, and invasion, further affecting tumor behavior (3).

In cancer metastasis, miRNAs facilitate the invasiveness of the tumor by regulating various cellular activities that include migration, invasion, proliferation, epithelial-mesenchymal transition (EMT), angiogenesis, and apoptosis (4). Their inherent stability underlines their use in the verification and validation of biomarkers (4). Moreover, miRNAs indeed represent therapeutic possibilities, especially in personalized cancer management (4). The role of miRNAs in drug resistance development has also been largely investigated, and its most prominent example is tamoxifen-resistant breast cancer (5). In this way, the deeper understanding of miRNA-mediated regulation in cancer could uncover novel diagnostic and therapeutic possibilities (6).

Colorectal cancer (CRC) is among the most common gastrointestinal malignancies worldwide, and early diagnosis is an important factor in reducing mortality. In recent years, miRNAs have shown great promise in this area. Dysregulated miRNA expression is closely associated with CRC initiation and progression (7). These miRNAs serve as biomarkers for early detection and are also integral to prognosis assessment and therapeutic decision-making (8). For instance, elevated serum levels of miR-21 and miR-92a have been identified as potential early detection biomarkers for CRC (9, 10). Specifically, miR-21 functions as an oncogenic miRNA, promoting tumor proliferation, invasion, and metastasis through the regulation of multiple target genes and signaling pathways (8, 11). Additionally, miR-92a correlates with various clinical features of CRC, highlighting its diagnostic potential (12, 13). Monitoring miRNA expression could thus provide novel strategies for early CRC diagnosis (7, 14).

miRNAs have also garnered significant attention for their roles in various other cancers. In malignant brain tumors, elevated miR-21 levels are linked to tumor growth and resistance to apoptosis, while the miR-34 family exerts tumor-suppressive effects (15). Similarly, in leukemia, miRNAs such as miR-125b are associated with poor prognosis, while miR-155 (16) and miR-181 (17) contribute to chemotherapy resistance (18). In prostate cancer, miR-145 and miR-221 regulate tumor invasion and prognosis (19). Furthermore, miRNA expression profiles offer valuable insights into patient responses to treatment, advancing the development of personalized therapies.

These four cancer types were selected due to their clinical significance and diverse representation of cancer biology. Colorectal and prostate cancers are among the most prevalent and well-studied cancers globally, with high morbidity and mortality rates, making them critical for understanding miRNA’s role in epithelial malignancies. In colorectal cancer (CRC), dysregulated miRNAs have been linked to tumorigenesis, with specific miRNAs acting as oncogenes or tumor suppressors (20). In prostate cancer, the role of miRNAs is equally significant. For instance, miR-137 has been identified as a critical regulator of hypoxia-mediated migration and epithelial-to-mesenchymal transition (EMT) in prostate cancer cells, highlighting its potential as a therapeutic target (21). Leukemia, as a representative of hematologic malignancies, offers insights into the regulatory functions of miRNA in blood and immune cell dysregulation. MicroRNAs (miRNAs) are crucial post-transcriptional regulators that play significant roles in the development and function of various immune cells, including those involved in hematological malignancies such as leukemia. For instance, miRNAs have been shown to influence the differentiation and function of natural killer (NK) cells, which are vital for early host defenses against tumors and infections (22). Brain tumors, particularly glioblastoma, highlight miRNA’s potential in addressing challenges in neurological cancers characterized by their aggressive nature and limited treatment options. For instance, specific miRNAs such as miR-21 and miR-34 have been shown to influence critical signaling pathways involved in cell proliferation, apoptosis, and differentiation, which are essential in the context of brain tumors (23). This selection ensures a comprehensive analysis of miRNA’s role across distinct cancer types, encompassing solid tumors, blood cancers, and malignancies with unique microenvironments and clinical challenges.

As research on miRNAs and cancer continues to progress, the rapid expansion of related literature presents a challenge for researchers to systematically summarize and evaluate these studies. Bibliometric analysis offers a valuable solution, enabling the identification of high-impact research, leading institutions, core journals, and key collaborative networks. It reveals research hotspots and future trends in fields such as colorectal cancer, brain tumors, leukemia, and prostate cancer. Additionally, bibliometric analysis can quantify the research focus and developmental trajectory of specific miRNAs across different cancer types, providing guidance for future studies. For instance, analyzing the study frequency of miR-21 and miR-145 across various cancers and their potential applications could highlight their diagnostic or therapeutic prospects. The interplay between the two is particularly noteworthy, as they exhibit antagonistic relationships in some cancer contexts, influencing cell behavior and treatment responses (24, 25). Besides, bibliometric analysis will identify gaps in knowledge, underlining the further need to explore miRNAs in particular subtypes of cancer. While the profound role of miRNAs has been unraveled in cancer diagnostics and treatments, bibliometric analysis traces progress within global research, optimizes the use of resources, and ensures that due attention is accorded to emerging topics. Finally, bibliometric analysis advances a systematic and holistic grasp of the place of miRNAs in cancer research.

## 2 Methods

### 2.1 Data collection

Literature published between 2014 and 2024 was identified from key databases, focusing on four significant cancer types: colorectal cancer, prostate cancer, leukemia, and brain tumors. These cancers were chosen for their clinical significance and the extensive research involving microRNA (miRNA) in their pathophysiology. To ensure a comprehensive analysis, the search strategy combined miRNA-related terms, including “MicroRNAs,” “Micro RNA,” “MicroRNA,” “miRNA,” and “miRNAs,” with cancer-specific terms tailored for each cancer type. For colorectal cancer, terms such as “Colorectal Neoplasms” and “Colorectal Cancer” were used; for prostate cancer, terms included “Prostatic Neoplasms” and “Prostate Cancer”; for leukemia, the search incorporated terms like “Leukemia” and “Leucocythaemia”; and for brain tumors, an extensive list was applied, including “Brain Neoplasms” and “Primary Brain Tumors.” Boolean operators (AND) were employed to combine miRNA-related terms with cancer-specific terms, ensuring a focused dataset for each cancer type. The search targeted journal articles, reviews, and conference papers to cover high-impact studies comprehensively. The detailed search strategy is provided in **Appendix 1**.

### 2.2 Bibliometric indicators

The main bibliometric indicators, taken into account for this study, are four: volume of literature, which refers to the total number of publications each cancer type has; citation count, which is a measure of academic impact at the publication level; impact factor (IF), which calculates reputation and influence of journals based on the average citation rates; and keyword burst detection, a method for detecting emerging trends in research or significant breakthroughs by means of detecting keywords with notable growth in their citation during a certain period.

### 2.3 Burst detection

Through the use of bibliometric software, burst detection analysis of keywords traces trends and emerging topics in research. The strength of each burst was calculated, with the start and end years recorded. This analysis pinpointed key areas where academic interest surged during specific periods, such as gene expression profiling, the role of microRNA in cancer progression, and extracellular vesicles in the tumor microenvironment.

### 2.4 Visualization and collaboration network analysis

Co-citation and keyword co-occurrence networks were created to examine collaboration patterns among authors, institutions, and countries. These visual maps identified the leading research groups and nations in cancer research. Co-citation analysis of the journal has also identified those core journals that have substantial academic influence in each area of cancer research.

### 2.5 Data analysis tools

The data in bibliometrics was processed and analyzed by using specialized tools like VOSviewer and CiteSpace, which are designed for visualizing a citation network and discovering the trending of research. The result would be presented in some tables and figures in country, institution, and journal rankings. They also provide an insight into the emerging directions identified through burst detection.

## 3 Results

**Figure 1:**
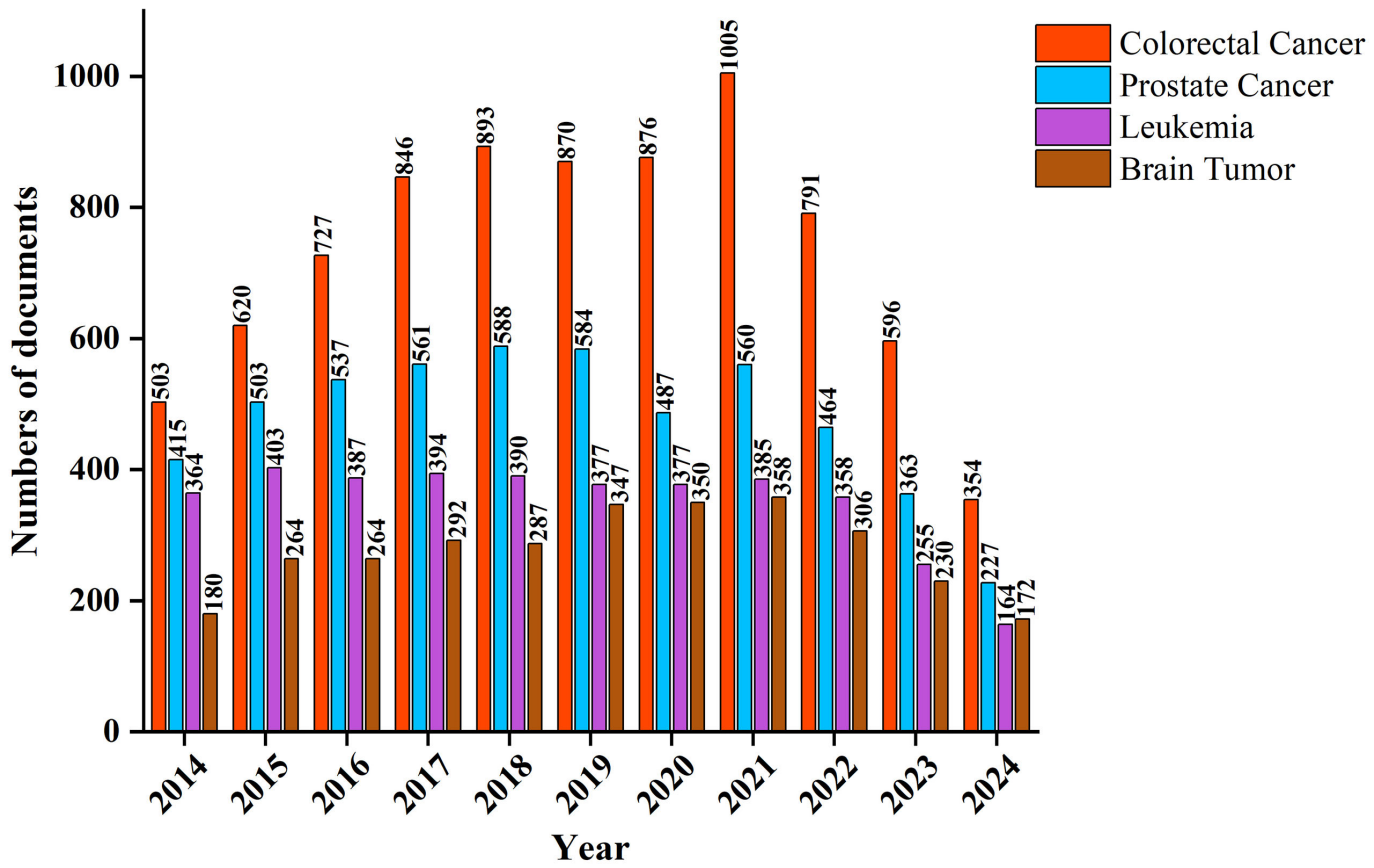
Bibliometric analysis of microRNA-related research publications in colorectal cancer, prostate cancer, leukemia, and brain tumor (2014–2024).

The final count was 5,524 articles on leukemia, 9,153 on colorectal cancer, 3,658 on brain tumors, and 6,466 on prostate cancer. **Figure 1** illustrates a systematic review focusing on the relationship of microRNA (miRNA) with four types of cancers: colorectal, prostate, leukemia, and brain tumors, from 2014 to 2024. There is erosion of interest in miRNA research for some of these malignancies. Colorectal cancer was thus, by the data, the highest regarding publications, starting with a steady rise from 2014 to a high of 1,005 papers in the year 2021. This steady rise suggests that miRNA involvement in colorectal cancer has become a prime area of research during the last ten years, with its magnitude significant between 2019 and 2021. It is likely that such a surge goes forward impelled by discoveries about the potential of miRNA regarding the diagnosis, prognosis, and treatments of colorectal cancer, thus giving more credence to its stature as a major research priority. Prostate cancer is second regarding the number of publications. While there was a progressive increase in the number of publications between 2014 and 2021, reaching a peak of 561 papers, it then rapidly declined to 227 papers by 2024. Although there is some sustained interest in the miRNA-prostate cancer relationship, the fluctuation in output indicates a reduction in enthusiasm compared with colorectal cancer. This leveling off may reflect changes in priorities, resource allocation, or other external factors affecting the evolution of miRNA research in prostate cancer.

Compared to colorectal and prostate cancer, miRNA research in leukemia has been relatively limited. In 2014, 264 papers on miRNA-leukemia were published, but decreased in 2016 and further reduced to a level of 164 by 2024. This difference is impressive considering the high value of leukemia in research among hematologic malignancies. Although miRNA represents an important area of cancer research, much more work has focused on its role in solid tumors compared with leukemia, reflecting a decided imbalance in both the emphasis of research efforts and investment of resources. It is possible that the concentration of research on solid tumors has eclipsed miRNA research in leukemia, perhaps leading to a poor division of labor across tumor types. Among the four cancer types, research on miRNA and brain tumors has produced the fewest publications. In 2014, only 180 papers were dedicated to miRNA-brain tumor associations. Although the number of publications fluctuated, a peak of 358 papers was reached in 2022. This increase was short-lived, however, as publication numbers dropped sharply to 172 by 2024. This pattern suggests that, despite brain tumors being major malignancies of the central nervous system with significant clinical demand, miRNA research in this area has not maintained a consistent momentum. The limited allocation of research resources may be contributing to the relatively low volume of studies compared to other cancer types.

**Table 1** illustrates that China has demonstrated exceptional performance in both the volume of publications and citation counts, particularly in colorectal and prostate cancer research, with 5,241 and 2,782 publications, respectively, ranking first globally. Notably, the United States, Germany, and Italy have also achieved significant results across various cancer research domains. Leading institutions, such as Nanjing Medical University, Shanghai Jiao Tong University, and Sun Yat-sen University, have contributed a substantial number of high-impact publications. In the realm of brain tumor and leukemia research, American institutions, including Ohio State University and the University of Texas MD Anderson Cancer Center, stand out for their notable contributions. Overall, cancer research is becoming increasingly concentrated in Chinese and American institutions, reflecting the dominant global influence these nations exert in the field.

**Table 1:** Top 10 countries/regions and organizations related to colorectal cancer, prostate cancer, leukemia, and brain tumor.

Type	Rank	Country	Avg. pub. year	Documents	Citations	Rank	Orgnization	Avg. pub. year	Documents	Citations
Colorectal cancer	1	Peoples R China	2018	5241	179986	1	Nanjing Med Univ	2018	308	12766
	2	Usa	2016	1219	86158	2	Shanghai Jiao Tong Univ	2017	203	15164
	3	Iran	2020	509	11549	3	Sun Yat Sen Univ	2017	199	10481
	4	Italy	2017	445	19789	4	Fudan Univ	2017	183	9763
	5	Japan	2016	404	25643	5	Southern Med Univ	2018	172	7343
	6	Germany	2016	299	27017	6	Zhengzhou Univ	2019	169	5101
	7	England	2017	219	8934	7	China Med Univ	2018	159	4942
	8	Spain	2017	218	12367	8	Zhejiang Univ	2018	145	5993
	9	South Korea	2017	198	6880	9	Soochow Univ	2018	140	4647
	10	India	2020	191	4014	10	Shandong Univ	2017	129	5055
Prostate cancer	1	Peoples R China	2018	2782	98393	1	Shanghai Jiao Tong Univ	2017	150	5849
	2	Usa	2015	1582	119511	2	Nanjing Med Univ	2017	142	6014
	3	Germany	2016	341	20399	3	Sun Yat Sen Univ	2017	128	5711
	4	Italy	2017	333	20523	4	Fudan Univ	2017	105	4674
	5	Iran	2020	234	5312	5	Univ Texas Md Anderson Canc Ctr	2015	94	9493
	6	Japan	2015	228	16355	6	China Med Univ	2017	90	3230
	7	England	2017	209	9599	7	Shandong Univ	2017	89	3398
	8	India	2019	205	5905	8	Univ Toronto	2015	87	3948
	9	Canada	2016	194	11055	9	Chinese Acad Sci	2017	86	5017
	10	Australia	2016	192	9595	10	Huazhong Univ Sci & Technol	2017	83	3911
Leukemia	1	Peoples R China	2017	2026	60362	1	Ohio State Univ	2012	290	45624
	2	Usa	2014	1500	146464	2	Univ Texas Md Anderson Canc Ctr	2013	148	15674
	3	Italy	2014	461	51215	3	Sun Yat Sen Univ	2017	101	5872
	4	Germany	2014	378	18873	4	Harvard Univ	2012	82	15821
	5	Iran	2019	250	3787	5	Huazhong Univ Sci & Technol	2017	81	2194
	6	Japan	2014	205	10023	6	Shanghai Jiao Tong Univ	2016	77	2504
	7	England	2015	178	8463	7	Nanjing Med Univ	2016	75	2655
	8	Spain	2014	165	7328	8	Univ Chicago	2012	74	10843
	9	Canada	2015	153	11578	9	Univ Ferrara	2012	70	23763
	10	India	2018	132	3340	10	Soochow Univ	2017	64	2548
Brain tumor	1	Peoples R China	2018	1680	51933	1	Nanjing Med Univ	2017	95	3424
	2	Usa	2016	920	68441	2	China Med Univ	2018	73	3052
	3	Italy	2017	216	11126	3	Harbin Med Univ	2018	70	2238
	4	Germany	2016	191	9984	4	Fudan Univ	2016	67	2616
	5	Iran	2021	144	2959	5	Sun Yat Sen Univ	2017	65	3939
	6	India	2019	140	3868	6	Capital Med Univ	2017	62	2653
	7	Canada	2016	116	6385	7	Shanghai Jiao Tong Univ	2017	58	2633
	8	England	2017	114	6737	8	Cent South Univ	2019	57	1252
	9	South Korea	2017	90	2987	9	Harvard Univ	2012	57	14748
	10	Japan	2016	88	5202	10	Soochow Univ	2017	51	1701

**Figure 2:**
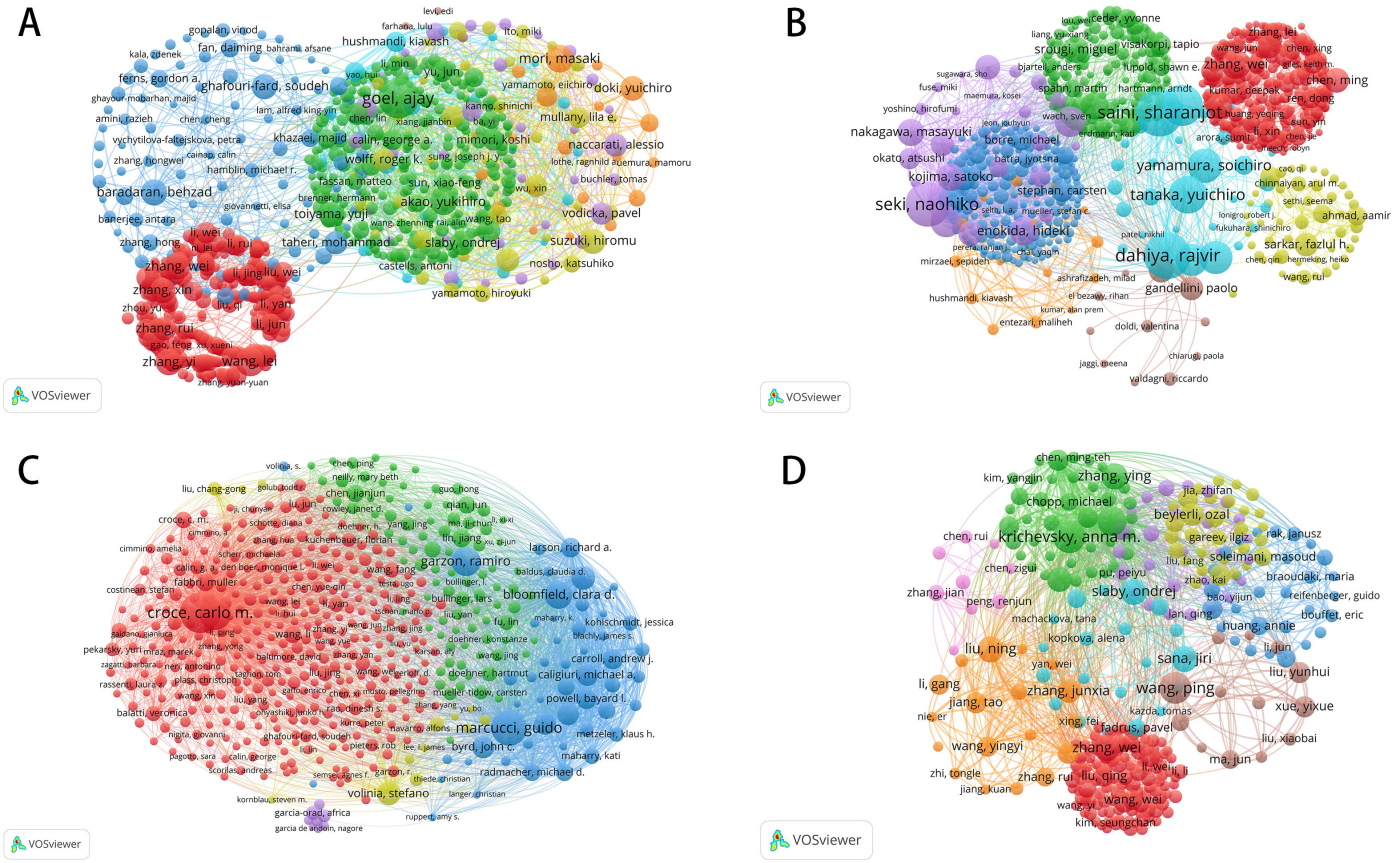
Bibliographic coupling networks of authors in miRNA research across four cancer types. **(A)** Bibliographic Coupling Network for Colorectal Cancer miRNA Research; **(B)** Bibliographic Coupling Network for Prostate Cancer miRNA Research; **(C)** Bibliographic Coupling Network for Leukemia miRNA Research; **(D)** Bibliographic Coupling Network for Brain Tumor miRNA Research.

Based on author coupling analysis of miRNA-related research across these cancer domains (**Figure 2**), the current research landscape, collaboration networks, and developmental trends were examined in detail. The size, color, and connections of the nodes in the map help to identify major research teams, authors with significant academic influence, and emerging research hotspots. In colorectal cancer, miRNA-related studies show the highest publication volume, with the most intricate collaboration networks among authors. **Figure 2A** highlights several highly interconnected research groups, particularly the green network centered around Ajay Goel, which illustrates his authoritative position in this field. The presence of other large-scale collaborative groups underscores the widespread interest in miRNA’s role in colorectal cancer, particularly as a regulatory factor in tumorigenesis and progression, and as a potential diagnostic and therapeutic biomarker. Since 2014, research on miRNA and colorectal cancer has grown steadily, peaking between 2019 and 2021, emphasizing its central importance in cancer research. The increasing density of these collaboration networks reflects a growing trend of interdisciplinary cooperation, further advancing the depth and scope of research in this area.

Although the volume of research on prostate cancer is not as extensive as that on colorectal cancer, the author coupling analysis in **Figure 2B** reveals a notable collaboration network. In particular, the research teams led by Sharanjot Saini and Rajvir Dahiya have established themselves as dominant forces in this field. However, the overall collaboration network for prostate cancer remains relatively fragmented, with weaker links between research groups. This indicates that miRNA research in prostate cancer has yet to reach the highly concentrated collaborative framework seen in colorectal cancer. Despite this, significant breakthroughs have been achieved, particularly in the discovery of biomarkers, disease prognosis evaluation, and the development of targeted therapies for prostate cancer, offering promising applications in personalized medicine. Moving forward, it will be essential to strengthen interdisciplinary and international collaboration to enhance overall research efficiency and impact. Research on miRNA in leukemia exhibits characteristics that are markedly distinct from the previous two cancer types. **Figure 2C** reveals a highly concentrated literature coupling network, with a red cluster centered around Carlo M. Croce, indicating that a small number of highly influential teams dominate research in this field. Despite the relatively lower volume of publications on leukemia, these core researchers and their teams have made substantial contributions, particularly in elucidating the mechanisms through which miRNA regulates the development and progression of the disease. Additionally, miRNA’s potential applications in the diagnosis and treatment of leukemia have been systematically explored. While this highly centralized collaboration network reflects the solid foundation of leukemia research, further expansion is needed to increase the breadth and diversity of studies in this area.

Compared to the other three cancers, miRNA research in brain tumors is still at a relatively early stage of development. **Figure 2D** shows that the author coupling network in this field is smaller in scale, with a more fragmented collaboration network. While research teams led by Zhang Wei and Ondrej Slaby have made notable contributions, the overall volume of miRNA research remains limited, and the density of collaboration is low. This may be due to the biological complexity of brain tumors and limitations in research funding and resources. Nonetheless, early findings on miRNA’s role in regulating the tumor microenvironment and tumor growth highlight the potential of this research. As more researchers join this field, the scale of studies and collaboration networks is expected to expand, potentially driving future breakthroughs in understanding and treating brain tumors.

**Figure 3:**
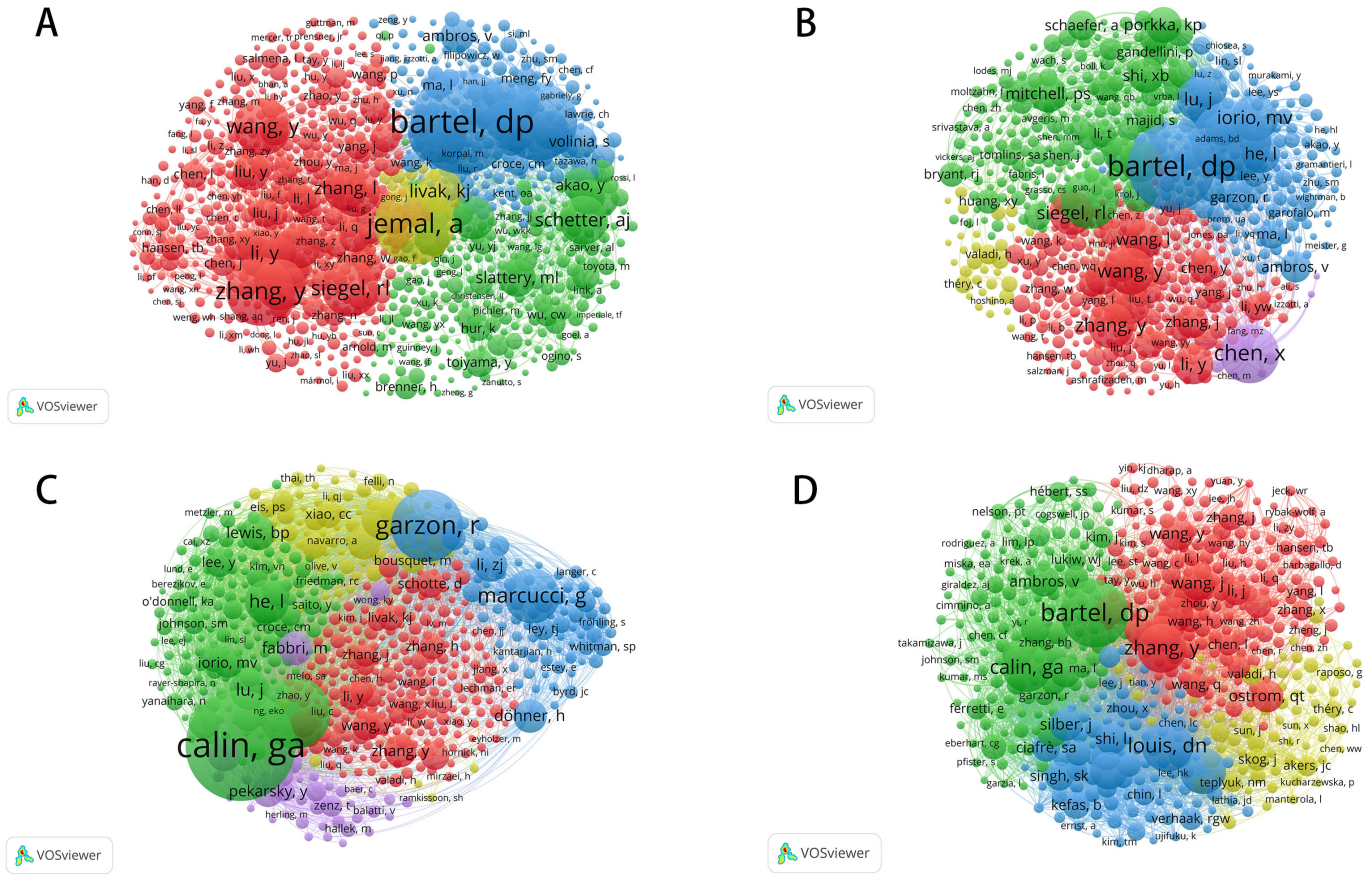
Co-citation networks of authors in miRNA research across four cancer types. **(A)** Co-Citation Network for Colorectal Cancer miRNA Research; **(B)** Co-Citation Network for Prostate Cancer miRNA Research; **(C)** Co-Citation Network for Leukemia miRNA Research; **(D)** Co-Citation Network for Brain Tumor miRNA Research.

**Figure 3** and **Table 2** provide a systematic analysis of co-cited authors, mapping the relationships among researchers studying microRNA (miRNA) across four cancer types: colorectal, prostate, leukemia, and brain tumors. These co-citation networks not only identify the most influential scholars but also reveal key research hotspots, emerging trends, and collaborative partnerships.

**Table 2:** Top 10 authors and co-cited authors related to colorectal cancer, prostate cancer, leukemia, and brain tumor.

Type	Rank	Author	Avg. pub. year	Documents	Citations	Rank	Co-cited author	Citations
Colorectal cancer	1	Goel, Ajay	2015	79	5931	1	Bartel, Dp	2153
	2	Wang, Lei	2017	46	2125	2	Jemal, A	1566
	3	Mori, Masaki	2015	42	3359	3	Calin, Ga	1537
	4	Akao, Yukihiro	2016	40	2052	4	Zhang, Y	1296
	5	Zhang, Yi	2017	40	1537	5	Chen, X	1061
	6	Baradaran, Behzad	2020	39	1573	6	Wang, Y	1030
	7	Zhang, Wei	2018	39	1488	7	Siegel, Rl	1012
	8	Ghafouri-Fard, Soudeh	2021	36	686	8	Li, J	892
	9	Suzuki, Hiromu	2015	36	2088	9	Li, Y	885
	10	Toiyama, Yuji	2016	36	2810	10	Schetter, Aj	845
Prostate cancer	1	Saini, Sharanjot	2016	62	2938	1	Bartel, Dp	1442
	2	Dahiya, Rajvir	2014	61	3293	2	Calin, Ga	1290
	3	Seki, Naohiko	2015	58	3119	3	Chen, X	878
	4	Majid, Shahana	2014	52	2889	4	Jemal, A	872
	5	Tanaka, Yuichiro	2014	46	2637	5	Wang, Y	659
	6	Yamamura, Soichiro	2014	40	2390	6	Iorio, Mv	654
	7	Shahryari, Varahram	2014	39	2150	7	Zhang, Y	644
	8	Deng, Guoren	2013	37	2194	8	Siegel, Rl	616
	9	Goto, Yusuke	2016	36	1583	9	Lu, J	595
	10	Ichikawa, Tomohiko	2015	35	1592	10	Liu, C	572
Leukemia	1	Croce, Carlo M.	2012	98	17411	1	Calin, Ga	2589
	2	Marcucci, Guido	2012	79	11214	2	Garzon, R	1421
	3	Calin, George A.	2012	64	10190	3	Bartel, Dp	1419
	4	Garzon, Ramiro	2012	56	8303	4	Marcucci, G	955
	5	Bloomfield, Clara D.	2012	52	6585	5	He, L	695
	6	Mrozek, Krzysztof	2012	40	4673	6	Lu, J	616
	7	Volinia, Stefano	2011	40	6848	7	O’Connell, Rm	567
	8	Byrd, John C.	2015	36	2454	8	Lewis, Bp	505
	9	Caligiuri, Michael A.	2012	35	4244	9	Chen, Cz	483
	10	Kolitz, Jonathan E.	2013	34	4205	10	Li, Zj	482
Brain tumor	1	Krichevsky, Anna M.	2013	23	8708	1	Bartel, Dp	847
	2	Wang, Ping	2016	22	1033	2	Louis, Dn	565
	3	Abounader, Roger	2014	20	2053	3	Zhang, Y	537
	4	Liu, Ning	2016	17	647	4	Calin, Ga	536
	5	Sana, Jiri	2018	17	370	5	Stupp, R	486
	6	Slaby, Ondrej	2018	17	370	6	Godlewski, J	366
	7	Zhang, Wei	2017	17	574	7	Li, Y	343
	8	Zhang, Ying	2016	17	1192	8	Zhang, L	322
	9	Purow, Benjamin	2012	16	2165	9	Wang, J	313
	10	Zhang, Junxia	2016	15	907	10	Wang, Y	311

In colorectal cancer research, miRNA studies demonstrate high academic activity and concentrated collaboration. Co-citation analysis identifies Bartel, DP as one of the most frequently cited scholars, whose work on miRNA’s role in colorectal cancer has profoundly shaped the field. The red cluster centered around Wang, Y, and Zhang, Y, points to the crucial contribution these authors have made in establishing not only miRNA’s regulatory role in tumor initiation and progression but also its potential as a biomarker. Meanwhile, the green cluster, featuring Schetter, AJ, and Slattery, ML, underlines their influence in the exploration of the diagnostic and therapeutic application of miRNA. Another very important contributor is Goel, Ajay, who has shared an immense contribution with a total of 79 publications and 5,931 citations. Collectively, these researchers have fostered a close-knit academic network with frequent collaborations. The primary focus of miRNA research in colorectal cancer remains on its regulatory mechanisms in tumor biology and its clinical application potential.

Although miRNA research in prostate cancer has developed a collaborative network, it remains less concentrated than in colorectal cancer. Co-citation analysis shows that Bartel, DP still plays a significant role in this field. However, the research landscape is more fragmented, characterized by several relatively independent academic groups. The red cluster, centered on Wang, Y, and Zhang, Y, highlights their crucial contributions to biomarker development and disease progression mechanisms. At the same time, Mitchell, PS, and Bryant, RJ, represented in the green cluster, focus primarily on the application of miRNA in non-invasive diagnostics. Among the prominent figures, Saini, Sharanjot, with 62 publications and 2,938 citations, stands out as a leader in miRNA research related to prostate cancer. While the collaborative network remains somewhat loose, miRNA research shows significant promise as a diagnostic tool and therapeutic target, with future efforts likely moving towards more cohesive collaboration. miRNA research in leukemia is largely driven by a small group of core scholars. Co-citation analysis reveals that Garzon, R, and Marcucci, G dominate the field, focusing on miRNA’s role as a regulatory factor in hematologic malignancies and its potential as a therapeutic target. Similarly, the green cluster, led by Calin, GA, has made significant contributions, particularly in exploring how miRNA influences the proliferation and differentiation of leukemia cells. Among the leading scholars, Croce, Carlo M., with 98 publications and 17,411 citations, stands out for his pivotal contributions to leukemia research. Overall, research on miRNA in leukemia has converged molecular directions, and there is close collaboration among key scholars. This model has benefited the translation of miRNA in the clinical applications of leukemia. Compared to other cancer types, miRNA research in brain tumors is still at an early stage of development, characterized by a more fragmented collaborative network. While Bartel, DP remains a central figure in the field, showcasing his broad influence, the red cluster, centered on Wang, Y, and Zhang, Y, highlights their contributions to studying miRNA’s regulatory role in tumor cell growth and differentiation. Meanwhile, the yellow cluster, featuring Calin, GA, and Ostrom, QT, focuses on miRNA’s potential applications in brain tumor diagnostics and therapy. Another influential figure in this area is Krichevsky, Anna M., with 23 publications and 8,708 citations. Although the miRNA-brain tumor research network is less concentrated than in other cancer fields, the research directions are becoming increasingly clear. With continued collaboration, miRNA’s role in the pathophysiology of brain tumors is expected to be further elucidated.

**Figure 4:**
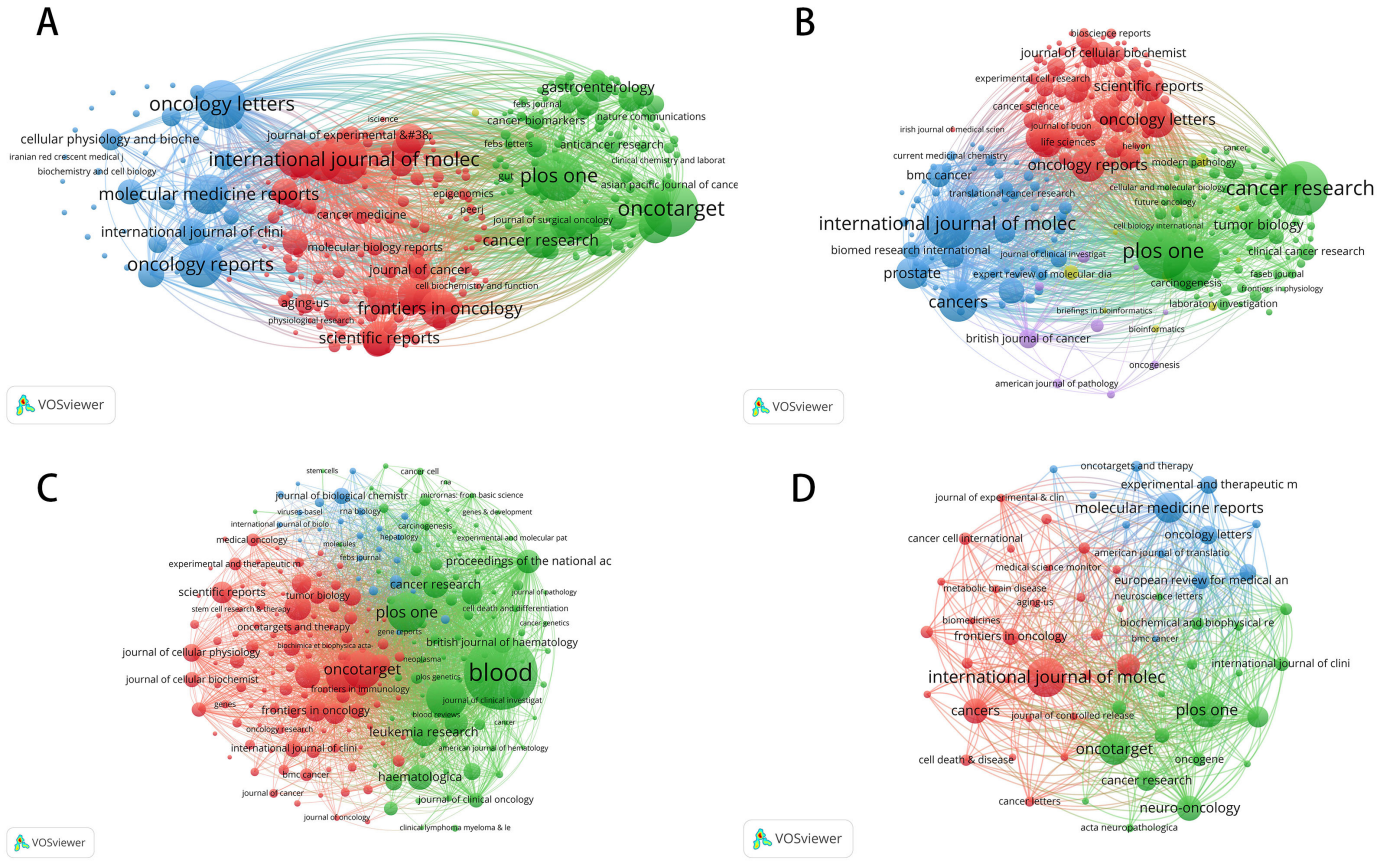
Bibliographic coupling networks of journals in miRNA research across four cancer types. **(A)** Bibliographic Coupling Network for Colorectal Cancer miRNA Research; **(B)** Bibliographic Coupling Network for Prostate Cancer miRNA Research; **(C)** Bibliographic Coupling Network for Leukemia miRNA Research; **(D)** Bibliographic Coupling Network for Brain Tumor miRNA Research.

**Figure 4** and **Table 3** analyze citation patterns across various academic journals, revealing key research hotspots, emerging trends, and the impact of different journals in advancing miRNA (microRNA) research. By coupling literature sources, the analysis highlights miRNA’s significant role in colorectal cancer, prostate cancer, leukemia, and brain tumor research, while also showcasing the contributions of key journals in these areas.

**Table 3:** Top 10 journals and co-cited journals related to colorectal cancer, prostate cancer, leukemia, and brain tumor.

Type	Rank	Journal	Documents	Citations	IF	JCR	Rank	Co-cited journal	Citations	IF	JCR
Colorectal cancer	1	Oncotarget	264	13581	5.168	Q2	1	Plos One	14366	2.9	Q1
	2	Plos One	221	12936	2.9	Q1	2	Cancer Res	14285	12.5	Q1
	3	International Journal Of Molecular Sciences	217	6688	4.9	Q2	3	Oncotarget	12348	5.168	Q2
	4	Oncology Letters	217	4507	2.5	Q3	4	Cell	11266	45.5	Q1
	5	Oncology Reports	188	6182	3.8	Q2	5	Oncogene	10267	6.9	Q1
	6	Cancers	159	3523	4.5	Q1	6	Nature	9988	50.5	Q1
	7	Molecular Medicine Reports	154	2938	3.4	Q2	7	P Natl Acad Sci Usa	9285	9.4	Q1
	8	Frontiers In Oncology	149	2660	3.5	Q2	8	Mol Cancer	7118	27.7	Q1
	9	Oncotargets And Therapy	142	3519	2.7	Q3	9	Clin Cancer Res	6996	10	Q1
	10	Cancer Research	124	6794	12.5	Q1	10	Nucleic Acids Res	6745	16.6	Q1
Prostate cancer	1	Plos One	197	12820	2.9	Q1	1	Cancer Res	16513	12.5	Q1
	2	Cancer Research	196	8919	12.5	Q1	2	Plos One	11747	2.9	Q1
	3	Oncotarget	173	9934	5.168	Q2	3	Oncogene	9602	6.9	Q1
	4	International Journal Of Molecular Sciences	160	5164	4.9	Q1	4	P Natl Acad Sci Usa	9348	9.4	Q1
	5	Cancers	120	2590	4.5	Q1	5	Cell	8587	45.5	Q1
	6	Oncology Letters	100	1924	2.5	Q3	6	Nature	8206	50.5	Q1
	7	Oncology Reports	94	3435	3.8	Q2	7	Oncotarget	8024	5.168	Q2
	8	Molecular Medicine Reports	88	1784	3.4	Q2	8	J Biol Chem	6112	4	Q2
	9	Prostate	86	5168	2.6	Q2	9	Nucleic Acids Res	5730	16.6	Q1
	10	Oncogene	84	9136	6.9	Q1	10	Clin Cancer Res	5591	10	Q1
Leukemia	1	Blood	315	15756	21	Q1	1	Blood	21123	21	Q1
	2	Leukemia	131	7513	12.8	Q1	2	P Natl Acad Sci Usa	12862	9.4	Q1
	3	Plos One	127	5066	2.9	Q1	3	Cell	9161	45.5	Q1
	4	Oncotarget	122	4813	5.168	Q2	4	Nature	9157	50.5	Q1
	5	International Journal Of Molecular Sciences	97	2330	4.9	Q1	5	Cancer Res	9052	5.168	Q1
	6	Leukemia Research	75	2104	2.1	Q3	6	Leukemia	7538	12.8	Q1
	7	Molecular Medicine Reports	70	1084	3.4	Q2	7	Plos One	6731	2.9	Q1
	8	Cancer Research	66	8134	12.5	Q1	8	Oncogene	6323	6.9	Q1
	9	Haematologica	65	664	8.2	Q1	9	Science	4792	44.7	Q1
	10	Oncology Letters	64	1059	2.5	Q3	10	J Biol Chem	4664	4	Q2
Brain tumor	1	International Journal Of Molecular Sciences	104	3271	4.9	Q1	1	Cancer Res	6248	12.5	Q1
	2	Plos One	87	4837	2.9	Q1	2	Plos One	5860	2.9	Q1
	3	Oncotarget	77	4104	5.168	Q2	3	Nature	5577	50.5	Q1
	4	Molecular Medicine Reports	73	1399	3.4	Q2	4	P Natl Acad Sci Usa	4965	9.4	Q1
	5	Neuro-Oncology	59	3041	16.4	Q1	5	Cell	4947	45.5	Q1
	6	Cancers	58	1367	4.5	Q1	6	Oncotarget	3759	5.168	Q2
	7	Scientific Reports	51	1379	3.8	Q1	7	Oncogene	3077	6.9	Q1
	8	Journal Of Neuro-Oncology	48	2147	3.2	Q2	8	J Biol Chem	3020	4	Q2
	9	Cancer Research	45	7077	12.5	Q1	9	Neuro-Oncology	2995	16.4	Q1
	10	Oncology Letters	41	979	2.5	Q3	10	Nucleic Acids Res	2965	16.6	Q1

In colorectal cancer research, miRNA has developed into a highly concentrated academic network, with a few key journals leading the way. The International Journal of Molecular Sciences, Oncotarget, and PLOS One are among the most frequently cited, emphasizing their importance as primary outlets for this research. Much of the work centers on miRNA’s role as a biomarker, particularly in tumor microenvironment regulation, early diagnosis, and personalized therapy. Oncotarget leads with 264 publications and 13,581 citations, with an impact factor of 5.168 and a ranking in the first quartile (Q1) of the JCR. PLOS One is similarly influential, with 221 publications and 12,936 citations. In contrast, the network of miRNA research in prostate cancer is more dispersed, within which several high-impact journals make significant contributions. The International Journal of Molecular Sciences has been central in the publishing of key findings on the role of miRNA as a biomarker of prognosis and therapeutic response. PLOS One has focused on non-invasive diagnostic technologies, concerned particularly with the detection of miRNA in blood and urine. Furthermore, the publishing of research letters in Oncology Letters has also contributed to the translational clinical research applications of miRNAs. Generally, miRNA research in prostate cancer is progressively moving toward clinical practice and has great potential to influence future personalized treatment strategies.

In leukemia studies, miRNA research is very concentrated, and the field is dominated by a few core journals. Among these, there are two of the most powerful journals: Blood and Leukemia. These two journals place great emphasis on the regulatory functions of miRNA in the proliferation, differentiation, and apoptosis of leukemia cells and have driven increasing interest in miRNA as a target for novel leukemia therapy. Thus, Blood has issued 315 articles altogether that have reached the level of 15,756 citations, establishing the journal’s position among the most influential ones in leukemia research. In contrast, miRNA research in brain tumors is still in its early stages, although it has grown rapidly in recent years. Oncotarget and PLOS One have actively published significant research on miRNA’s role in regulating brain tumor cell growth, differentiation, and invasiveness, particularly in relation to glioblastoma, a highly malignant tumor. Similarly, journals like Neuro-Oncology and Cancer Research have contributed to exploring miRNA’s mechanisms, especially concerning the tumor microenvironment and treatment response. While miRNA research in brain tumors has yet to form a highly concentrated network, it is expected to consolidate further as research in this area deepens.

**Figure 5:**
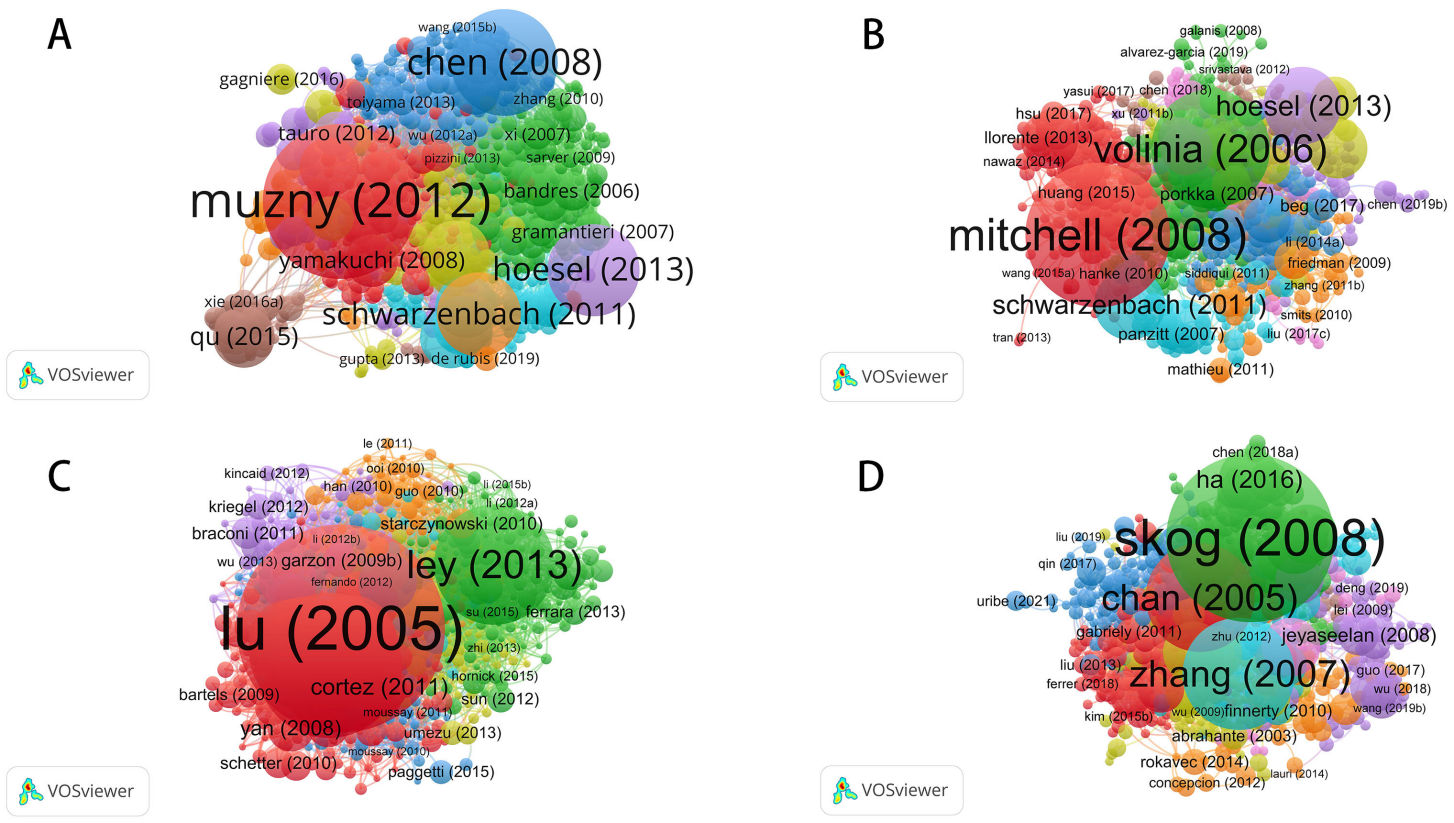
Citation networks of key miRNA research publications across different cancer types. **(A)** Citation Network for Colorectal Cancer miRNA Research; **(B)** Citation Network for Prostate Cancer miRNA Research; **(C)** Citation Network for Leukemia miRNA Research; **(D)** Citation Network for Brain Tumor miRNA Research.

An analysis of highly cited literature reveals research hotspots, emerging trends, and the clinical potential of miRNA across various cancer types (**Figure 5**; **Table 4**). The following sections examine miRNA’s role in colorectal, prostate, leukemia, and brain tumors.

**Table 4:** Top 10 references related to colorectal cancer, prostate cancer, leukemia, and brain tumor.

Type	Rank	Literature	Title	DOI	Source	IF/JCR	Citations
Colorectal cancer	1	Muzny (2012)	Comprehensive molecular characterization of human colonand rectalcancer	https://doi.org/10.1038/nature11252	Nature	50.5/Q1	5216
	2	Chen (2008)	Characterization of microRNAs in serum: a novel class of biomarkers for diagnosis of cancer and other diseases	https://doi.org/10.1038/cr.2008.282	Cell Research	28.1/Q1	3056
	3	Schwarzenbach (2011)	Cell-free nucleic acids as biomarkers in cancer patients	https://doi.org/10.1038/nrc3066	Nature Reviews Cancer	72.5/Q1	2169
	4	Asangani (2008)	MicroRNA-21 (miR-21) post-transcriptionally downregulates tumor suppressor Pdcd4 and stimulates invasion, intravasation and metastasis in colorectal cancer	https://doi.org/10.1038/sj.onc.1210856	Oncogene	6.9/Q1	1566
	5	Burk (2008)	A reciprocal repression between ZEB1 and members of the miR-200 family promotes EMT and invasion in cancer cells	https://doi.org/10.1038/embor.2008.74	EMBO Reports	6.5/Q1	1416
	6	Qu (2015b)	Circular RNA: A new star of noncoding RNAs	https://doi.org/10.1016/j.canlet.2015.06.003	Cancer Letters	9.1/Q1	1403
	7	Wellner (2009)	TheEMT-activator ZEB1 promotes tumorigenicity by repressing stemness-inhibiting microRNAs	https://doi.org/10.1038/ncb1998	Nature Cell Biology	17.3/Q1	1392
	8	Korpal (2008)	The miR-200 family inhibits epithelial-mesenchymal transition and cancer cell migration by direct targeting of E-cadherin transcriptional repressors ZEB1 and ZEB2	https://doi.org/10.1074/jbc.c800074200	Journal of Biological Chemistry	4.0/Q2	1328
	9	Yu (2017b)	Fusobacterium nucleatum Promotes Chemoresistance to Colorectal Cancer by Modulating Autophagy	https://doi.org/10.1016/j.cell.2017.07.008	Cell	45.5/Q1	1311
	10	Yamakuchi (2008)	miR-34a repression of SIRT1 regulates apoptosis	https://doi.org/10.1073/pnas.0801613105	Proceedings of the National Academy of Sciences	9.4/Q1	1115
Prostate cancer	1	Mitchell (2008)	Circulating microRNAs as stable blood-based markers for cancer detection	https://doi.org/10.1073/pnas.0804549105	Proceedings of the National Academy of Sciences	9.4/Q1	6488
	2	Volinia (2006)	A microRNA expression signature of human solid tumors defines cancer gene targets	https://doi.org/10.1073/pnas.0510565103	Proceedings of the National Academy of Sciences	9.4/Q1	4782
	3	Schwarzenbach (2011)	Cell-free nucleic acids as biomarkers in cancer patients	https://doi.org/10.1038/nrc3066	Nature Reviews Cancer	72.5/Q1	2169
	4	Gao (2009)	c-Myc suppression of miR-23a/b enhances mitochondrial glutaminase expression and glutamine metabolism	https://doi.org/10.1038/nature07823	Nature	50.5/Q1	1672
	5	Kosaka (2010)	Secretory Mechanisms and Intercellular Transfer of MicroRNAs in Living Cells	https://doi.org/10.1074/jbc.m110.107821	Journal of Biological Chemistry	4.0/Q2	1550
	6	Gibb (2011)	The functional role of long non-coding RNA in human carcinomas	https://doi.org/10.1186/1476-4598-10-38	Molecular Cancer	27.7/Q1	1447
	7	Anastasiadou (2018)	Non-coding RNA networks in cancer	https://doi.org/10.1038/nrc.2017.99	Nature Reviews Cancer	72.5/Q1	1350
	8	Becker (2016)	Extracellular Vesicles in Cancer: Cell-to-Cell Mediators of Metastasis	https://doi.org/10.1016/j.ccell.2016.10.009	Cancer Cell	48.8/Q1	1209
	9	Liu (2011c)	The microRNA miR-34a inhibits prostate cancer stem cells and metastasis by directly repressing CD44	https://doi.org/10.1038/nm.2284	Nature Medicine	58.7/Q1	1156
	10	Vo (2019)	The Landscape of Circular RNA in Cancer	https://doi.org/10.1016/j.cell.2018.12.021	Cell	45.5/Q1	1129
Leukemia	1	Lu (2005)	MicroRNA expression profiles classify human cancers	https://doi.org/10.1038/nature03702	Nature	50.5/Q1	7996
	2	Volinia (2006)	A microRNA expression signature of human solid tumors defines cancer gene targets	https://doi.org/10.1073/pnas.0510565103	Proceedings of the National Academy of Sciences	9.4/Q1	4782
	3	Calin (2002)	Frequent deletions and down-regulation of micro- RNA genes miR15 and miR16 at 13q14 in chronic lymphocytic leukemia	https://doi.org/10.1073/pnas.242606799	Proceedings of the National Academy of Sciences	9.4/Q1	3970
	4	Ley (2013)	Genomic and Epigenomic Landscapes of Adult *De Novo* Acute Myeloid Leukemia	https://doi.org/10.1056/nejmoa1301689	The New England Journal of Medicine	96.2/Q1	3655
	5	Iorio (2005)	MicroRNA Gene Expression Deregulation in Human Breast Cancer	https://doi.org/10.1158/0008-5472.can-05-1783	Cancer Research	12.5/Q1	3347
	6	Calin (2004b)	Human microRNA genes are frequently located at fragile sites and genomic regions involved in cancers	https://doi.org/10.1073/pnas.0307323101	Proceedings of the National Academy of Sciences	9.4/Q1	3340
	7	Cimmino (2005)	miR-15 and miR-16 induce apoptosis by targeting BCL2	https://doi.org/10.1073/pnas.0506654102	Proceedings of the National Academy of Sciences	9.4/Q1	2833
	8	Croce (2009)	Causes and consequences of microRNA dysregulation in cancer	https://doi.org/10.1038/nrg2634	Nature Reviews Genetics	39.1/Q1	2545
	9	Zhang (2007)	microRNAs as oncogenes and tumor suppressors	https://doi.org/10.1016/j.ydbio.2006.08.028	Developmental Biology	2.5/Q2	2177
	10	Calin (2005)	A MicroRNA Signature Associated with Prognosis and Progression in Chronic Lymphocytic Leukemia	https://doi.org/10.1056/nejmoa050995	The New England Journal of Medicine	96.2/Q1	1913
Brain tumor	1	Skog (2008)	Glioblastoma microvesicles transport RNA and proteins that promote tumour growth and provide diagnostic biomarkers	https://doi.org/10.1038/ncb1800	Nature Cell Biology	17.3/Q1	3876
	2	Zhang (2007)	microRNAs as oncogenes and tumor suppressors	https://doi.org/10.1016/j.ydbio.2006.08.028	Developmental Biology	2.5/Q2	2177
	3	Chan (2005)	MicroRNA-21 Is an Antiapoptotic Factor in Human Glioblastoma Cells	https://doi.org/10.1158/0008-5472.can-05-0137	Cancer Research	12.5/Q1	2087
	4	Ha (2016)	Exosomes as therapeutic drug carriers and delivery vehicles across biological membranes: current perspectives and future challenges	https://doi.org/10.1016/j.apsb.2016.02.001	Acta pharmaceutica Sinica.B	14.7/Q1	937
	5	Ciafrè (2005)	Extensive modulation of a set of microRNAs in primary glioblastoma	https://doi.org/10.1016/j.bbrc.2005.07.030	Biochemical and Biophysical Research Communications	2.5/Q3	914
	6	Zhang (2015a)	Microenvironment-induced PTEN loss by exosomal microRNA primes brain metastasis outgrowth	https://doi.org/10.1038/nature15376	Nature	50.5/Q1	906
	7	Fong (2015)	Breast-cancer-secreted miR-122 reprograms glucose metabolism in premetastatic niche to promote metastasis	https://doi.org/10.1038/ncb3094	Nature Cell Biology	17.3/Q1	867
	8	Liang (2007)	Characterization of microRNA expression profiles in normal human tissues	https://doi.org/10.1186/1471-2164-8-166	BMC Genomics	3.5/Q2	866
	9	Krichevsky (2009)	miR-21: a small multi-faceted RNA	https://doi.org/10.1111/j.1582-4934.2008.00556.x	Journal of celluar and molecular medicine	4.3/Q2	816
	10	Silber (2008)	miR-124 and miR-137 inhibit proliferation of glioblastoma multiforme cells and induce differentiation of brain tumor stem cells	https://doi.org/10.1186/1741-7015-6-14	BMC Medicine	7.0/Q1	776

In colorectal cancer research, the citation network identifies studies by Muzny (2012) (5,216 citations) and Schwarzenbach (2011) (2,169 citations) as central works, primarily focusing on miRNA’s role as an early diagnostic biomarker and its function in tumor biology. MiRNA plays a pivotal role in regulating colorectal cancer cell proliferation, migration, and invasion, making it a valuable tool for diagnosis and a promising therapeutic target. Similarly, studies by Chen (2008) (3,056 citations) and Yamakuchi (2008) (1,115 citations) delve further into miRNA’s regulatory functions in cancer progression, highlighting its potential not only for diagnostics but also for guiding treatment decisions. High citation rates of these studies indicate the maturity and importance of miRNA research in the field. These works represent the citation cores: Mitchell (2008), with 6,488 citations, and Volinia (2006), with 4,782 citations, focus on miRNA expression patterns in prostate cancer and their critical roles in the initiation and progression of cancer. These articles emphasize miRNA as a biomarker with good potential for early detection and prognosis. Schwarzenbach (2011) (2,169 citations) further supports miRNA utility in non-invasive diagnostics by blood and urine testing, offering new diagnostic possibilities for early prostate cancer screening in a minimally invasive way. Additional studies by Porkka (2007) and Hoesel (2013) explore miRNA’s role in personalized therapies, particularly in the development of novel treatment strategies. Together, this body of research underscores miRNA’s significant potential not only as a diagnostic tool but also as a therapeutic agent.

In leukemia research, Lu (2005) (7,996 citations) and Ley (2013) (3,655 citations) are the most cited studies, reflecting broad recognition of miRNA’s pivotal role in leukemia cell regulation. Lu (2005) first uncovered miRNA’s regulatory function in leukemia cells, laying the groundwork for subsequent research. Ley (2013) further explores miRNA’s therapeutic potential, particularly in overcoming drug resistance and improving treatment response. Building on these foundational studies, Cortez (2011) and Garzon (2009b) focus on the clinical translation of miRNA into leukemia treatment, providing strong evidence for the development of personalized therapies in the future. In brain tumor research, Skog (2008) (3,876 citations) and Chan (2005) (2,087 citations) are the most cited studies, focusing on miRNA’s role in brain tumors, particularly gliomas. MiRNA is not only key in regulating tumor cell proliferation, differentiation, and migration, but also holds promise as a therapeutic target. Zhang (2007) (2,177 citations) and Ha (2016) (937 citations) further explore miRNA’s mechanisms within the tumor microenvironment, suggesting that miRNA’s clinical applications in brain tumor treatment are rapidly expanding, especially with the development of new therapies. The high citation rates of these studies highlight miRNA as an emerging research focus in brain tumor studies.

**Figure 6:**
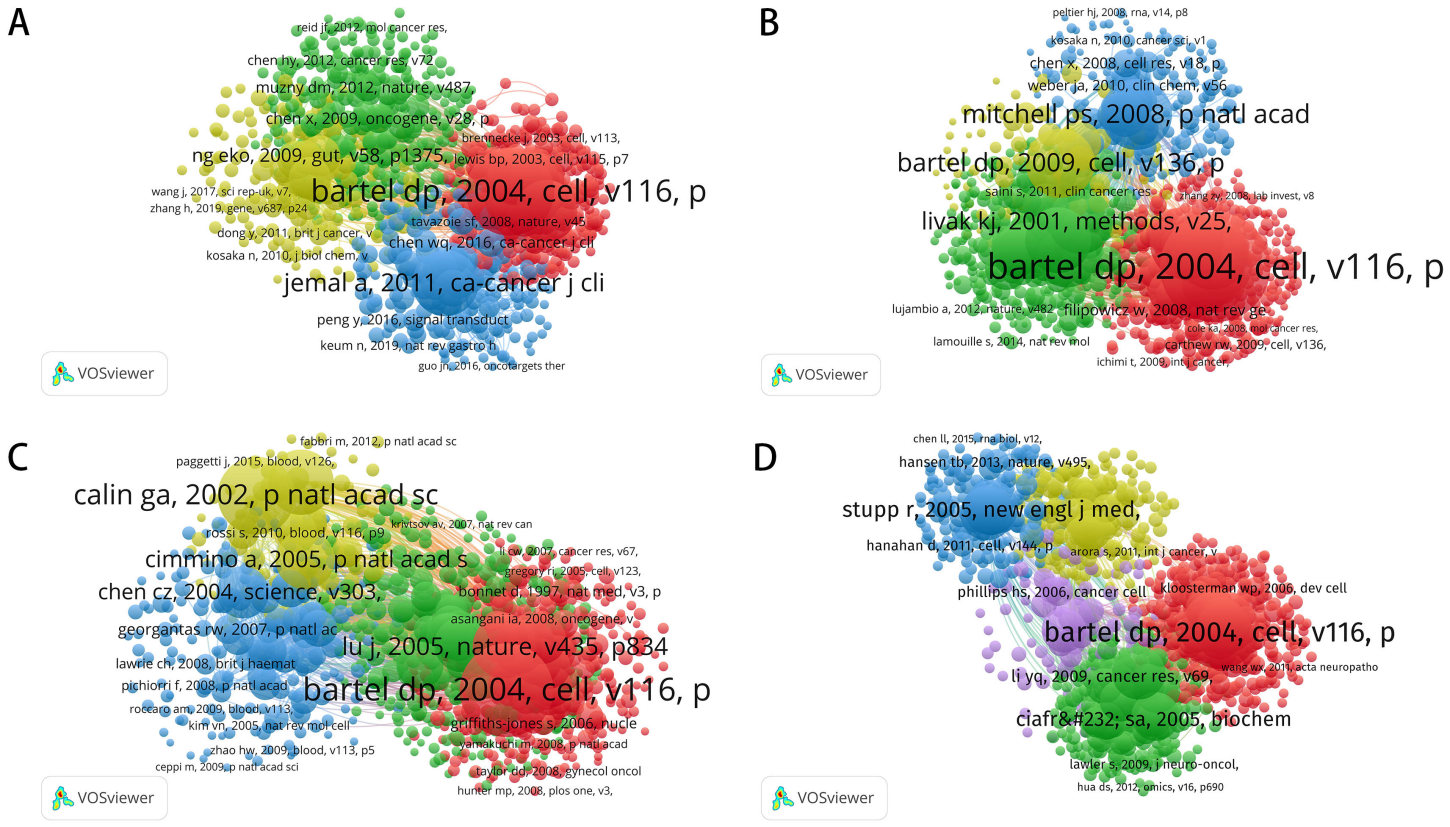
Co-citation analysis of key references in miRNA research across cancer types. **(A)** Co-Citation Network in Colorectal Cancer miRNA Research; **(B)** Co-Citation Network in Prostate Cancer miRNA Research; **(C)** Co-Citation Network in Leukemia miRNA Research; **(D)** Co-Citation Network in Brain Tumor miRNA Research.

The co-citation analysis of colorectal cancer underscores Bartel’s broad influence on miRNA function, while Ng Eko (2009) and Chen (2009) clarify miRNA’s regulatory mechanisms in tumorigenesis and cancer progression. In prostate cancer research, the seminal works of Mitchell (2008) and Volinia (2006) reveal miRNA’s crucial role in diagnosis and prognosis, while Gao (2009) focuses on its regulatory role in tumor progression.In leukemia, studies by Calin (2002) and Chen (2004) uncover miRNA’s pathogenic mechanisms in hematologic malignancies, while Lu (2005) and Cimmino (2005) offer further insights into its roles in cell cycle regulation and signal transduction. In brain tumors, Stupp (2005) and Phillips (2006) highlight miRNA’s regulatory role in gliomas, while Bartel (2004) offers theoretical support for understanding its function in nervous system tumors. Overall, miRNA, as a key regulator of gene expression, has demonstrated great potential as both a biomarker and therapeutic target across multiple cancers, paving the way for future advancements in cancer diagnosis and treatment (**Figure 6**).

**Figure 7:**
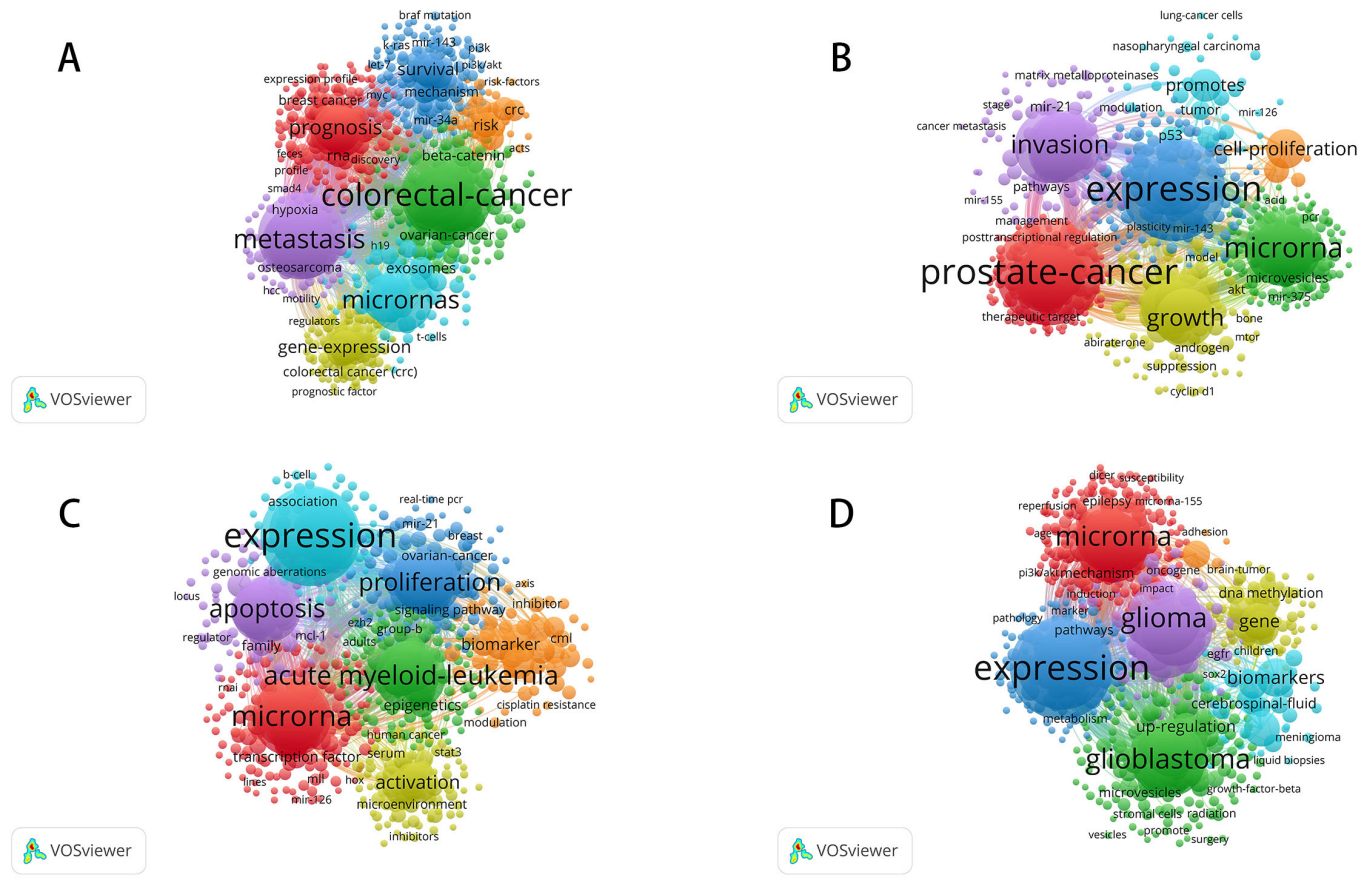
Research hotspots and trends of miRNA in various cancer types. **(A)** Keyword Co-occurrence Network of miRNA-Related Research in Colorectal Cancer; **(B)** Keyword Co-occurrence Network of miRNA-Related Research in Prostate Cancer; **(C)** Keyword Co-occurrence Network of miRNA-Related Research in Leukemia; **(D)** Keyword Co-occurrence Network of miRNA-Related Research in Brain Tumors.

**Figure 8:**
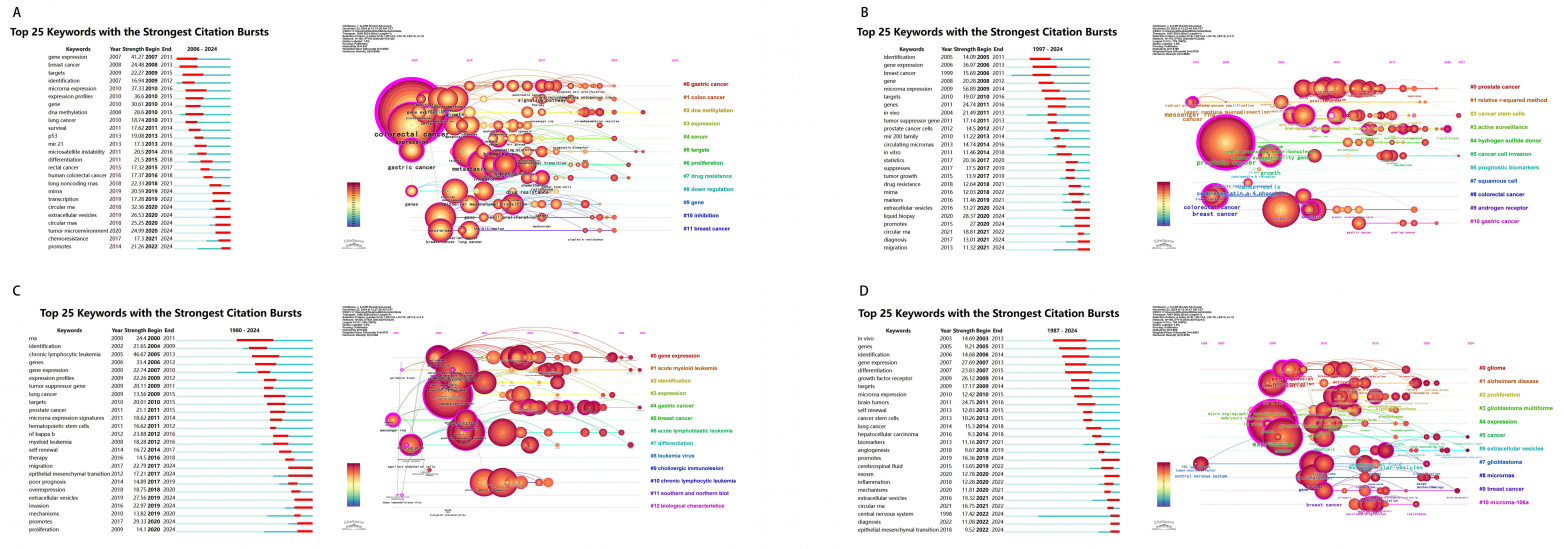
Dynamic trends in microRNA-related research: Top 25 keywords with the strongest citation bursts across colorectal cancer, prostate cancer, leukemia, and brain tumors. **(A)** Top 25 Keywords with Strongest Citation Bursts in Colorectal Cancer Research; **(B)** Top 25 Keywords with Strongest Citation Bursts in Prostate Cancer Research; **(C)**Top 25 Keywords with Strongest Citation Bursts in Leukemia Research; **(D)**Top 25 Keywords with Strongest Citation Bursts in Brain Tumor Research.

The bibliometric analysis offers valuable insights into significant trends and emerging research hotspots across colorectal cancer, prostate cancer, leukemia, and brain tumors. As shown in **Figures 7**, **8**, the co-citation networks and keyword citation bursts highlight dynamic shifts in research focus over time for each cancer type. For colorectal cancer (**Figures 7A**, **8A**), prominent keywords such as “microRNA,” “prognosis,” and “metastasis” underscore a strong focus on miRNA’s role in gene regulation and disease progression. Early citation bursts highlight “expression profiles” (burst strength 16.09, 2014–2015) and “microRNA expression” (burst strength 14.69, 2014–2016) as central to foundational studies, while recent attention has shifted to “extracellular vesicles” (burst strength 15.09, 2020–2024) and “tumor microenvironment” (burst strength 12.27, 2020–2024), emphasizing intercellular communication and its relevance for diagnostics and therapeutics. In prostate cancer (**Figures 7B**, **8B**), the co-citation network centers on keywords like “microRNA,” “invasion,” and “growth,” reflecting a strong interest in miRNA’s impact on tumor proliferation and metastasis. Early research prioritized “microRNA expression” (burst strength 14.73, 2014–2015) for its relevance in diagnostics and prognosis, while recent trends focus on “extracellular vesicles” (burst strength 15.45, 2020–2024) and the emerging role of “circular RNAs” (burst strength 13.64, 2020–2024), expanding the scope of molecular targets in cancer progression. Leukemia research (**Figures 7C**, **8C**) highlights keywords such as “microRNA,” “acute myeloid leukemia,” and “proliferation,” demonstrating a focus on hematological malignancies. Early studies (2014–2015) emphasized “chronic lymphocytic leukemia” (burst strength 9.75) and “tumor suppressor genes” (burst strength 6.87) to understand miRNA’s regulatory role, whereas more recent attention has shifted to “circular RNA” (burst strength 10.6, 2019–2024) and “non-coding RNA” (burst strength 7.67, 2020–2024), reflecting their growing importance as therapeutic targets. For brain tumors (**Figures 7D**, **8D**), the co-citation network emphasizes keywords like “microRNA,” “glioma,” and “glioblastoma,” underscoring miRNA’s central role in understanding the molecular biology of these aggressive cancers. Early bursts focused on “malignant glioma” (burst strength 6.98, 2014–2016) and “brain tumors” (burst strength 9.69, 2014–2016), while recent research highlights non-invasive diagnostic approaches with keywords like “liquid biopsy” (burst strength 7.36, 2020–2024) and “extracellular vesicles” (burst strength 7.63, 2022–2024), showcasing their clinical potential.

## 4 Discussion

This bibliometric analysis identified significant trends and emerging research hotspots in miRNA-related cancer studies, focusing on four major cancer types: colorectal cancer, prostate cancer, leukemia, and brain tumors. Key research areas identified include gene expression profiles, extracellular vesicles, and non-coding RNAs. Among these, colorectal cancer research led in publication volume, demonstrating sustained interest. In contrast, brain tumor research exhibited the least activity, though a recent rise in studies indicates growing attention. Prostate cancer research showed fluctuating interest, gradually shifting toward clinical applications of miRNA. Although leukemia studies were fewer in number, high-impact research by leading scholars underscored the critical role of miRNA in hematologic malignancies.

### 4.1 Implications for future clinical applications

Through a comprehensive analysis of miRNA research trends across various cancer types, this study provides valuable insights for future clinical applications, particularly in the realm of personalized medicine.

#### 4.1.1 Advancing miRNA-based precision medicine

Our study highlights the pivotal role of miRNA in colorectal cancer, prostate cancer, leukemia, and brain tumors, especially in the molecular mechanisms of tumor initiation, progression, metastasis, and drug resistance. These findings strongly support the application of miRNA in personalized cancer therapy. In colorectal cancer, miRNA’s well-established role as a tumor biomarker enables early diagnosis and guides treatment decisions. Moreover, our analysis reinforces miRNA’s significant interaction with the tumor microenvironment and intercellular communication, offering critical theoretical foundations for more precise, individualized therapeutic strategies.

#### 4.1.2 Prospects for miRNA in non-invasive diagnostics

Our analysis of liquid biopsies and extracellular vesicles demonstrates the broad potential of miRNA in non-invasive diagnostic technologies. Simple blood or urine tests that monitor miRNA expression offer convenient and accurate tools for early detection, treatment monitoring, and relapse prediction. The widespread adoption of these technologies reduces the risks associated with traditional tissue biopsies while improving patients’ quality of life. In particular, our research underscores the clinical feasibility of these techniques in prostate cancer and brain tumors, where liquid biopsies have already proven effective as diagnostic tools.

#### 4.1.3 Clinical potential of miRNA-targeted therapy

This study provides theoretical support for miRNA-targeted therapy by analyzing miRNA’s functional roles in various cancers. Our findings demonstrate that miRNA can serve both as a diagnostic biomarker and as a direct intervention in cancer progression by either restoring or inhibiting specific miRNA functions. For example, using miRNA mimics or anti-miRNA oligonucleotides (antagomirs) to target cancer-related miRNAs can effectively suppress tumor growth and overcome chemotherapy resistance. This analysis identifies potential therapeutic targets for future clinical trials, laying a solid foundation for the development of innovative miRNA-based therapies.

#### 4.1.4 Advancing combined therapies with miRNA

Our findings suggest that combining miRNA with traditional therapies such as chemotherapy, radiotherapy, and immunotherapy may yield significant synergistic effects. By modulating miRNAs involved in immune evasion, the tumor microenvironment, and genetic mutations, combined therapies could enhance the effectiveness of cancer treatments and reduce recurrence rates. For instance, miRNAs potential in immune checkpoint inhibitor therapies warrants further investigation, especially for patients unresponsive to existing treatments. Future clinical research can build on the miRNA hotspots identified in this study to develop more effective combination therapies.

#### 4.1.5 Accelerating the translation of miRNA from research to clinical practice

This study systematically maps miRNA research progress in cancer, providing a critical bridge from basic research to clinical practice. By identifying influential research hotspots and potential clinical applications, we offer scientific guidance for designing future clinical trials and developing miRNA-targeted drugs and diagnostic tools. This study highlights miRNA’s therapeutic potential and clinical feasibility, offering theoretical support for emerging miRNA therapies and paving the way for their rapid adoption in clinical practice.

### 4.2 Implications for future clinical research

This study systematically examines miRNA research trends across various cancer types, providing critical insights and guidance for future clinical research, with a particular focus on personalized medicine and the development of innovative therapeutic strategies.

Our findings underscore the pivotal role of miRNAs in cancer initiation, progression, and resistance to therapy, with a particular emphasis on their regulation of the tumor microenvironment. These insights offer a clear direction for clinical research, especially in investigating how modulating miRNA levels could improve treatment efficacy and mitigate resistance. Future research should prioritize elucidating the connection between miRNAs and resistance mechanisms, particularly in cancers that are resistant to chemotherapy or immunotherapy. Identifying key miRNA signaling pathways will empower researchers to design more effective interventions that enhance therapeutic outcomes.

Our research has shown that microRNAs (miRNAs) play an important role in the occurrence and development of cancers such as colorectal cancer, prostate cancer, leukemia, and brain tumors. A large number of studies have found that certain miRNAs are upregulated or downregulated in tumor tissues and are closely related to tumorigenesis, metastasis, and drug resistance. However, the results of these studies are not completely consistent and there are still some controversies. Some studies have indicated that the overexpression of specific miRNAs may be associated with a tumor-suppressive effect, while other studies have found that the overexpression of the same miRNAs may promote the proliferation and metastasis of tumor cells. Different experimental designs, sample sources, and data analysis methods may lead to these differences. To address the limitations of miRNA therapy, future research needs to focus on improving the targeting and specificity of miRNAs. Nanotechnology can be utilized to specifically deliver miRNA carriers to tumor sites, thereby reducing their impact on normal tissues. Moreover, by employing synthetic miRNAs or miRNA inhibitors along with specific delivery systems, it becomes possible to enhance the therapeutic effect and reduce side effects.

This study also points out the very important potential of miRNA in noninvasive diagnostic technologies, especially through liquid biopsy applications. Detection of miRNA in blood or urine may allow clinical research to confirm its clinical use as an early diagnostic tool and for real-time monitoring of treatment outcomes of cancer. This approach not only improves patient outcomes but also greatly reduces the need for invasive procedures. Our bibliometric analysis points out that liquid biopsy and exosomes are the main hotspots in research, which may provide a clear direction for future studies, especially in prostate cancer and brain tumors.

Moreover, this work underlines the great potentiality of miRNA in individualized therapy. The accumulation or down-regulation observed in different patients might have paramount importance in designing specific therapeutic approaches. The clinical use in the future might allow for a tailored treatment plan by optimization of therapies according to tumor subtype, genetic background, and changes in the tumor microenvironment. Thus, it enhances the precision of treatment and reduces the number of unnecessary interventions, thus minimizing the side effects.

Further, this work has underlined the mutual interactions of miRNAs with other molecular biomarkers, notably circular and long non-coding RNAs. Further investigation might be directed at these complex interactions and their integrated contributions to the process of tumor development, thus constituting a theoretical basis for the elaboration of more sophisticated biomarker systems that could allow for a more precise identification of cancer type and stage of its progression.

Finally, this study provides a basis for future multi-institutional and interdisciplinary collaborations. We give a guide for international collaboration by finding the important scholars and networks of collaborations in different areas of cancer research. Besides encouraging miRNA research cross-border cooperation, which will accelerate the medical clinical application, the progress will also be rapid with the sharing of data and resources. Upcoming clinical research should extend international collaboration, particularly in the development and validation of new miRNA-based therapeutic and diagnostic agents, to expedite the transformation of research findings into actual clinical practice.

### 4.3 Strengths and limitations

This bibliometric analysis synthetizes the key research hotspots in miRNA-related studies across different types of cancer, thereby yielding a clear roadmap for future research directions. Among the major strengths of the present study are the use of robust co-citation network and burst analysis tools, which pinpoint critical research areas and trends that will most likely shape the landscape of clinical treatments in the future. The analysis of literature from 2014 to 2024 will provide adequate evidence that the trends identified are timely and relevant, thus showing the increasing importance of miRNA in cancer studies. Besides, the application of multi-analysis methods provides an overall view related to the development of cancer and points to either the advance of basic research or the possibility of clinical applications. Such an integrated approach lays the foundation for further research on miRNA-based diagnostics and treatments. It also serves to highlight high-impact studies and leading scholars, providing a very valuable resource for novice researchers and teams seeking to make a future contribution in this field.

However, there are some limitations to the study. First, the information in this review will be supported only by published literature and, thus, may not reflect unpublished or newly emerging data that could dramatically alter research directions in the field of miRNA. Second, the analysis will be focused only on four tumor types, so important developments concerning other malignancies will not be taken into consideration. Also, the selected period, 2014-2024, may miss more updated findings or changes in their research focus outside of this period. And lastly, while the critical hotspots of research trend provided in this study are sound, due to the inability to elaborate on the molecular mechanism of miRNA, further detailed information on its clinical application could not be explored.

## 5 Conclusion

From this comprehensive bibliometric overview, we extracted some hotspots in research, trends, and clinical potentials of miRNA related to colorectal cancer, prostate cancer, leukemia, and brain tumors. miRNA performed extensively in early diagnosis, biomarker development, therapeutic monitoring, regulation of the tumor microenvironment, and intercellular communication; the broad potential is further underlined. With the development of non-invasive diagnostic technologies such as liquid biopsy and personalized treatment strategies, miRNA has great potential in clinical settings. Future studies are also needed to further develop the role of miRNA within a network of non-coding RNA and its interaction synergistically with other molecular markers in the elements of drug resistance, combination therapies, and immunotherapy. This study, while underlining the varied potential of miRNA, further depicts those clinical trials and international collaborations will become indispensable in accelerating the translation process from basic research to clinics. In summary, miRNA is emerging as a key in diagnostics and treatment, opening totally new perspectives in therapy and thus laying the foundation for advances in precision medicine.
