# Supplementary material for: Unveiling hotspots of emerging research in the miRNA-related mechanism underlying cancer through comprehensive bibliometric analysis with implications for precision medicine and non-invasive diagnostics
Source: Front Oncol. 2025 Jan 15;14:1521251. doi: 10.3389/fonc.2024.1521251 (PMC11774920; doi:10.3389/fonc.2024.1521251)
Supplement: Supplementary file 1 [file DataSheet1.docx]

Colorectal Neoplasms(9153)

#1

((((TS=(MicroRNAs )) OR TS=(Micro RNA)) OR TS=(MicroRNA)) OR TS=(miRNA)) OR TS=(miRNAs)

#2

(((((((TS=(Colorectal Neoplasms)) OR TS=(Colorectal Neoplasm)) OR TS=(Colorectal Tumors)) OR TS=(Colorectal Tumor)) OR TS=(Colorectal Cancer)) OR TS=(Colorectal Cancers)) OR TS=(Colorectal Carcinoma)) OR TS=(Colorectal Carcinomas)

#1 AND #2

Prostatic Neoplasms(6466)

#1

((((TS=(MicroRNAs )) OR TS=(Micro RNA)) OR TS=(MicroRNA)) OR TS=(miRNA)) OR TS=(miRNAs)

#2

(((((((((TS=(Prostatic Neoplasms)) OR TS=(Prostatic Neoplasm)) OR TS=(Prostate Neoplasms)) OR TS=(Prostate Neoplasm)) OR TS=(Prostate Cancer)) OR TS=(Prostate Cancers)) OR TS=(Cancer of Prostate)) OR TS=(Cancer of the Prostate)) OR TS=(Prostatic Cancer)) OR TS=(Prostatic Cancers)

#1 AND #2

Leukemia(5524)

#1

((((TS=(MicroRNAs )) OR TS=(Micro RNA)) OR TS=(MicroRNA)) OR TS=(miRNA)) OR TS=(miRNAs)

#2

(((((TS=(Leukemia)) OR TS=(Leukemias)) OR TS=(Leucocythaemia)) OR TS=(Leucocythaemias)) OR TS=(Leucocythemia)) OR TS=(Leucocythemias)

#1 AND #2

Brain Neoplasms(3658)

#1

((((TS=(MicroRNAs )) OR TS=(Micro RNA)) OR TS=(MicroRNA)) OR TS=(miRNA)) OR TS=(miRNAs)

#2

(((((((((((((((((((((((((((((TS=(Brain Neoplasms)) OR TS=(Brain Neoplasm)) OR TS=(Brain Tumors)) OR TS=(Brain Tumor)) OR TS=(Brain Cancer)) OR TS=(Brain Cancers)) OR TS=(Malignant Brain Neoplasm)) OR TS=(Malignant Brain Neoplasms)) OR TS=(Cancer of Brain)) OR TS=(Cancer of the Brain)) OR TS=(Brain Malignant Neoplasm)) OR TS=(Brain Malignant Neoplasms)) OR TS=(Malignant Primary Brain Tumors)) OR TS=(Malignant Primary Brain Neoplasms)) OR TS=(Primary Malignant Brain Neoplasms)) OR TS=(Primary Malignant Brain Tumors)) OR TS=(Intracranial Neoplasm)) OR TS=(Intracranial Neoplasms)) OR TS=(Brain Benign Neoplasm)) OR TS=(Brain Benign Neoplasms)) OR TS=(Benign Brain Neoplasm)) OR TS=(Benign Brain Neoplasms)) OR TS=(Primary Brain Tumor)) OR TS=(Primary Brain Tumors)) OR TS=(Primary Brain Neoplasms)) OR TS=(Primary Brain Neoplasm)) OR TS=(Recurrent Brain Tumor)) OR TS=(Recurrent Brain Tumors)) OR TS=(Brain Metastases)) OR TS=(Brain Metastase)

#1 AND #2
